# Histofluorescence analysis of Pittsburgh Compound B labeled pathology in Down syndrome putamen and frontal cortex

**DOI:** 10.1002/alz.091764

**Published:** 2025-01-03

**Authors:** Jeremy P. Rouanet, Kim Chan Ngo, Tamara M Shamas, Brenna Phelan, Ashley L. Fung, Michael J. Phelan, Jesse R. Pascual, Phong Ngo, Elizabeth J. Andrews, Sierra Wright, Florence Lai, Julia Kofler, Milos D Ikonomovic, Elizabeth Head, Alzheimer Biomarkers Consortium ‐ Down Syndrome (ABC‐DS)

**Affiliations:** ^1^ University of California, Irvine, Irvine, CA USA; ^2^ Harvard Medical School, Boston, MA USA; ^3^ Massachusetts General Hospital, Boston, MA USA; ^4^ University of Pittsburgh, Pittsburgh, PA USA; ^5^ VA Pittsburgh Healthcare System, Pittsburgh, PA USA; ^6^ The UC Irvine Institute for Memory Impairments and Neurological Disorders (UCI MIND), Irvine, CA USA

## Abstract

**Background:**

Brain deposits of amyloid‐β (Aβ), one of the hallmark pathologies of Alzheimer disease (AD), are consistently present in people with Down syndrome (DS) after the age of 30 years. Positron emission tomography (PET) radioligands like [3H]Pittsburgh Compound‐B (PiB) allow for visualizing Aβ accumulation in living people. In DS, the earliest and strongest PiB‐PET retention is in the striatum, differing from late‐onset AD. The neuropathological substrates of early PiB retention in DS striatum are unknown. The goal of this postmortem study is to compare histofluorescent labeling with cyano‐PiB, a fluorescent derivative of PiB, in the striatum and frontal cortex of people with DS.

**Method:**

Cyano‐PiB histofluorescence load (% area) was evaluated on postmortem tissue from putamen and frontal cortex (n = 43; age 42‐70 years, 24F/19M) using whole slide imaging and digital quantification. Cyano‐PiB histofluorescence was combined with Aβ immunofluorescence on a subset (n = 3) of cases.

**Result:**

After the age of 42 years, cyano‐PiB loads did not increase as a function of age at death in frontal cortex (layers II‐IV; p = 0.64) and putamen (p = 0.76). Cyano‐PiB loads were not greater in putamen compared to frontal cortex. Putamen and frontal cortex cyano‐PiB loads directly correlated (r = 0.52). Cyano‐PiB labeled plaques were immunolabelled with Aβ‐targeting antibodies 6E10, 12F4, and 4G8, in both putamen and frontal cortex, with less overlap observed in deep frontal cortical layers.

**Conclusion:**

Striatal cyano‐PiB load in putamen was not greater than in the frontal cortex layers II‐IV in people with DS above the age of 42 years. The partial overlap of cyano‐PiB labeled amyloid plaques with Aβ immunoreactive plaques suggests that only a subset of Aβ plaques were detected with cyano‐PiB. Further characterization of cyano‐PiB loads in deep layers of the frontal cortex as well as in DS cases at younger ages is warranted.

